# Strengthening emergency nursing education: A Pathway to Pakistan’s emergency care resilience

**DOI:** 10.12669/pjms.42.(ICON26).15696

**Published:** 2026-04

**Authors:** Tariq Aziz, Nasik Awan, Muhammad Wajid, Rashida Merchant

**Affiliations:** 1 Tariq Aziz, Nurse Manager, Nursing Services, Indus Hospital & Health Network, Karachi, Pakistan; 2 Nasik Awan, Senior Head Nurse, Nursing Services, Indus Hospital & Health Network, Karachi, Pakistan; 3 Muhammad Wajid, Senior Head Nurse, Nursing Services, Indus Hospital & Health Network, Karachi, Pakistan; 4 Rashida Merchant, Director, Nursing Services, Indus Hospital & Health Network, Karachi, Pakistan

The escalating burden of acute illness, trauma, and disaster-related emergencies in Pakistan necessitates a highly competent and adaptable emergency nursing workforce. Pakistan’s healthcare system is further challenged as a growing number of nurses migrate for better professional opportunities and security abroad.^1^ Emergency departments nationwide face prolonged waiting times, overcrowding, and limited resources, challenges that underscore the need for systematic investment in emergency nursing education.^2^ Pakistan needs about 700,000 nurses but had only 116,659 registered in 2020, leaving a massive workforce gap for a population of over 240 million.^1^ WHO guidelines mandate three nurses per doctor, but in Pakistan, there are only 0.5 nurse per doctor. A strong and structured approach to workforce development is essential for safeguarding patient outcomes, strengthening preparedness, and ensuring rapid, safe, and coordinated emergency care.

The Pakistan Nursing and Midwifery Council (PNMC) has taken important steps toward enhancing nursing capacity through standardized curricula, professional regulation, and support for continuing education.^3^ These reforms provide a foundation for competency based practice, however, evidence suggests that gaps remain in specialized emergency training, particularly in triage, trauma care, and disaster response.^4^ Published evaluations of emergency nursing curricula in South Asia highlight variability in clinical exposure, limited simulation training, and insufficient integration of international emergency care standards.^5^ These findings align with Pakistan’s needs and emphasize the importance of structured postgraduate preparation in emergency nursing.

In recent years, specialized training programs such as a one-year Post-Basic Diploma in Disaster & Emergency Nursing and Accident & Emergency Nursing diploma have contributed to skill development by combining theoretical instruction with supervised practice. Though these programs represent progress; published analyses indicate that their scale remains insufficient relative to national workforce demands, with graduates often reporting of limited opportunities for advanced specialization and leadership roles.^6^ Similarly, global collaborative courses such as WHO-ICRC Basic Emergency Care(BEC) & Collaborative Advance Trauma course(CAT-C) strengthen foundational competencies, but can not replace the need for sustained, locally contextualized, specialized education programs for emergency care workforce.

While several initiatives exist, there are persistent gaps requiring focused attention. First, Pakistan’s emergency nursing workforce remains numerically inadequate for the volume and acuity of emergency cases, reinforcing the need to expand enrollment capacity and create advanced career pathways. Second, retention challenges driven by burnout, heavy workloads, and limited professional recognition diminish long-term impact of training efforts. Third, the current evidence base shows a scarcity of nurse-led research in trauma and emergency care, limiting the development of context specific best practices. Last but not the least, emergency nurses are not formally integrated into national emergency care policy or disaster planning frameworks, despite their frontline expertise.

Addressing these issues requires a unified national strategy at country level which links national regulatory bodies (PNMC, HEC etc.) educational institutions, and healthcare systems. Strengthening simulation based training, introducing competency assessments aligned with international standards, and expanding postgraduate specialties can enhance clinical readiness. Likewise, formal career pathways, supportive working conditions, and research capacity building are critical for sustaining a resilient emergency care workforce.

## Authors Contribution:

**TA** Conceptualization, initial draft, and responsible for the study.

**NA MW:** Literature search, critical review

**RM:** Conceptualizing

All authors have read the final version of manuscript.
